# A Biocurator Perspective: Annotation at the Research Collaboratory for Structural Bioinformatics Protein Data Bank

**DOI:** 10.1371/journal.pcbi.0020099

**Published:** 2006-10-27

**Authors:** Kyle Burkhardt, Bohdan Schneider, Jeramia Ory

**Affiliations:** National Center for Biotechnology Information, United States of America

Like most scientists, annotators at the Research Collaboratory for Structural Bioinformatics (RCSB) (http://www.pdb.org) dread the immortal cocktail party question “So, what do you do?” Unlike for some jobs, however, their answer can leave *other scientists* at the party with no response. Even within the structural biology community, our job is not well-understood. Throughout this perspective, we will shed light on the daily challenges faced by annotators at the RCSB and give the reader a glimpse at the juggling act that defines the job of a biocurator.

## Acquisition of the Data

The Protein Data Bank (PDB) [1,2] was established at the US Atomic Energy Commission's Brookhaven National Laboratory in 1971 as the international repository for 3-D macromolecular data of protein and protein–nucleic acids complexes. It is one of few archival data repositories, and the structural biology community depends on it for file storage and access. In 1999, management of the PDB was assumed by the RCSB. In 2003, the collaboration among PDB deposition centers was formalized as the worldwide PDB (wwPDB) [3]. The wwPDB partners are the RCSB PDB in the United States, the Molecular Structure Database—European Bioinformatics Institute (MSD–EBI) in Hinxton, United Kingdom, and the Protein Data Bank—Japan (PDBj) in Osaka, Japan. While some biological databases are built on data derived from the primary literature, all of the wwPDB's data is directly submitted as a prerequisite to publication (a requirement of most journals). The primary data consist of atomic coordinates from the most common experimental techniques; X-ray crystallography, NMR spectroscopy, or electron microscopy. Additionally, authors may submit the experimental data used to solve the structure. Authors from around the world are continually submitting new data to the wwPDB. The average number of deposited structures is increasing steadily from more than 2,600 depositions in 1999 to an estimated 7,000 depositions for 2006. One major challenge is developing software and management strategies that will keep up with this growing data deposition rate.

At the RCSB PDB, authors are encouraged to validate their structures prior to deposition using a variety of tools provided online. These tools help authors find corresponding sequence data for their entries and point out potential errors in the coordinates. When satisfied with these initial checks, the author uploads the coordinates and experimental data into a web-based input tool. After entering all information required by the wwPDB and any additional information she or he wishes, the author finalizes the initial entry and receives an identification code, commonly called the PDB ID. Each structure in the PDB has a unique ID, and it is used in publications dealing with the structure. At this point, the author's initial responsibility for the entry is finished, though not for long. As outlined below, annotators work extensively with authors to ensure that the data represents the author's work in the best possible way.

## Annotation of the Data

### 

#### The staff.

The curation staff of the wwPDB personally annotates every incoming PDB entry. The 20 annotators worldwide bring a variety of experiences and expertise to the task at hand. The level of scientific training ranges from bachelor's degrees in chemistry and biology to postdoctoral experience in a particular research area relevant to structural biology, such as X-ray crystallography, NMR spectroscopy, or cryo-electron microscopy.

#### Initial annotation.

To someone not familiar with biological databases, biological curation might appear to be a data entry job. As anyone who has tried to use a biological database knows, however, this is simply not the case. A database's usefulness is an equal combination of the data going into it, how it is annotated, and its underlying design. The average user should be able to find data easily without sacrificing the ability to perform complex queries if necessary. While we do not control the quality of the data, thorough annotation and user-friendly database tools are the keys to making the database useful.

**Box 1. Annotators Work to Represent PDB Data in the Best Possible Way by:**

- Reviewing entry for self-consistency
- Matching given title to structure
- Correcting format errors in data and coordinates
- Checking sequence using BLAST [13]
- Inserting sequence database reference
- Providing protein name and synonyms
- Checking scientific name of the source organism
- Confirming chemical consistency between ligand name and the 3-D coordinates
- Adding information describing the biological assembly
- Checking entry visually
- Generating validation reports
- Finding citation references with PubMed [14]

At the RCSB, entries are processed from start to finish by the same annotator. This system has certain disadvantages but one essential advantage: personal responsibility. Annotators are also trained to curate data of all experimental types, regardless of their scientific background. Those with backgrounds in an area of specialization serve as a resource for the others within the annotation group. The three wwPDB centers communicate daily regarding curation issues, sharing knowledge, and serving as a resource for each other. An annotator's responsibility for a particular entry does not end with finishing the first pass through the system. The annotator is also responsible for releasing the entry and maintaining its updates and possible corrections. Annotators archive all author correspondence by entry ID. This archive is accessible by all annotators, and ensures that the data and all author communications for every entry is always retrievable, even if the annotator originally responsible for it has left the PDB.

The goal of annotation is to make each entry not only self-consistent but also consistent with the rest of the archive. To this end, annotators help authors represent their data in the best possible way. Annotators routinely review the incoming data and perform many standard inspections (see Box 1).

Frequently, annotators find inconsistencies in the data and work with the authors to correct these problems. Two types of problems are most common; discrepancies between the submitted sequence and coordinates, and incorrect description of small molecules (ligands). Problems with sequences include single or multiple mismatches between the deposited sequence, the sequence in the coordinates, and/or the sequence as it appears in the reference sequence database. Occasionally, entries are submitted where the sequence and coordinates do not match at all or are swapped between entities. Ligand problems can range from inverted chiral centers to a complete mismatch between the author's claim and the actual ligand coordinates. Human error is at the root of most other problems: authors accidentally upload the same coordinate set for multiple entries, or submit entries where information in the title, source, or refinement statistics does not match the contents of the entry. A separate source of problems arises when submitted experimental data have low or no correlation with the coordinates. It should be stressed at this point that while the structural data are thoroughly checked and potential problems are reported to the author, it is only the author who can actually modify the structure. Most authors update and correct problems when possible. Without annotator intervention, many errors would propagate through the database.

However trivial they might seem, formatting issues continue to be a problem. Most of these formatting issues and other inconsistencies are caught during the annotator's first review of the entry. In severe cases, annotation on the entry stops, and the author is contacted for further clarification; typically these cases result in the resubmission of a corrected coordinate set by the author. While mistakes such as switching coordinate sets are understandable, we continually work toward providing the best tools and resources possible to prevent mistakes. The RCSB PDB is constantly working to educate authors about common errors.

When the annotator is able to complete annotation of the entry, internal software generates the flat files, which are then returned to the author for review and approval. A validation report containing any concerns or questions the annotator had during the curation process is also attached.

It is important to note that the annotation staff does not judge the quality of the structure, and has little editorial control over what is released. Annotators often bring potential errors to the attention of the author, but it is ultimately up to the author to decide whether or not to address these issues. PDB users are encouraged to use software tools and their own knowledge to judge the quality of any given structure.

#### After initial annotation.

The PDB curation staff corresponds and collaborates with the author to ensure accurate representation of their data within the PDB entry. Structural data is unique. Authors should consider their entry as a representation of their scholarly work, though some view PDB deposition as an arduous formality that must be completed before their publication is accepted.

Communication between authors and annotators can be challenging and time-consuming. On occasion authors may send new data or provide new information, which necessitates reprocessing the entry. New coordinate sets may be sent for the same entry multiple times. If the coordinates are for a large or complex entry, this translates into many hours of redundant effort. A disproportionate amount of biocurator time can be spent on a select few entries requiring repeated reprocessing due to author-related delays. Complex, last-minute changes to entries after initial approval are frustrating for even the most seasoned annotator. Some authors can be unresponsive or uncooperative, thus impeding completion of their entry. This generally results in additional effort for the annotator as s/he reviews reports and sends reminders. The wwPDB plans to move to a more formal and structured communication protocol between author and curator to address many of these issues. We also encourage authors to review and analyze the information uploaded at the time of deposition to reduce mistakes.

Once approved by the author, entries are stored in the internal archive and released according the status set at time of deposition. The majority of authors choose to release their depositions upon publication of the primary citation. Depositions may be held until a year after deposition, including structures that are deposited as “hold until publication.” If, after a year, the citation has not been published, the authors must decide to either release or withdraw the entry. Software tools are used to search for structures that have been published and require annotator inspection for updating the citation and entry release. Weekly scripts list entries that are approaching the end of their hold period and need to be released. Reminder letters are sent to unresponsive authors if questions remain regarding the entry.

#### Typical day.

As authors deposit data 24/7, 365 days a year, the job of annotation essentially never ends. New entries are processed, and recently processed entries are updated with author-provided corrections, sometimes multiple times for the same entry. Trying to keep up with processing new structures, incorporating corrections for processed structures, and releasing structures can be taxing. It isn't uncommon to spend a significant amount of time processing a complex entry, only for the author to respond with a new coordinate set or to introduce additional details about the experimental data that changes the way the entry should be processed.

Most author–annotator interaction occurs through e-mail. If an annotator receives a phone call, it is rarely to compliment a job well done. Keeping up with the deluge of incoming e-mail represents dealing with more than 100 e-mail messages per day, ranging from updates about a specific entry to general questions. Answering generic questions is rotated among the staff, but keeping up with authors' demands for immediate response presents a challenge. Of all the members of the database staff, the annotators have the most frequent interaction with the community. Outreach is an integral part of annotator's work via e-mail exchange with authors and by attending national and international conferences.

At the RCSB PDB, staff meetings are held almost every week for continuing education, to discuss difficult or unusual cases, or to collaborate with the programming staff. Time is spent testing and retesting new deposition and annotation software tools, participating in brainstorming sessions, and making suggestions for procedural and software improvement. Frequent exchange meetings are held with the staff of the MSD–EBI group, and frequent e-mail exchange occurs between the RCSB and PDBj.

Stability of the annotation staff is essential to a productive team. When an annotator leaves his/her job, it impacts the entire staff, especially those who take over the processed entries of the outgoing annotator. Locating and interviewing possible new annotators is a time-consuming task. Finding the right combination of attitude, temperament, and scientific skills in one person can be daunting. The ideal annotator is someone who can multitask, has a pleasant, resilient personality, has a background in structural biology, and is a quick learner. The training period for new annotators can last anywhere from two to six months, depending on the person. Although one experienced annotator is dedicated to training new annotators, all annotators participate in training by answering questions and reviewing the new annotator's work.

#### Job satisfaction.

There are good benefits to the biocurator job. It provides opportunities for those with analytical skills who want to remain involved in their field but tire of the uncertainties associated with academic life. There is the ability to work from home if necessary, or even from a remote location. It can certainly be fun to see the hottest, latest structures during annotation and interact with the members of the structural biology community. Curating entries for inclusion in a resource that is used by so many people (10,000 individuals per day) also instills pride. For people who need to “get something done” every day, there is a unique sense of accomplishment after processing many entries in a particular day or week. Again, contrast this to the research lab, where days or weeks can go by with much hard work and few or little results.

On the other hand, the process of annotation is essentially the same for every structure. Reviewing all of the information associated with each structure can be tedious and monotonous. The pressure involved in maintaining the data rate can be distressing. Add to this all the duties previously mentioned, and the situation becomes a concentration challenge even for an experienced multitasker.

To combat the monotony of annotation, some annotators engage in other activities such as teaching, management, outreach, programming, or structural bioinformatics research. However, the reality is that because of the high data influx, curation plus another outlet can equate to two full-time jobs. It can be overwhelming, and burnout is a distinct possibility.

## Challenges

The main challenge ahead for PDB curators deals with the increasing volume and complexity of the data [4]. As volume increases, so do the difficulties associated with data formatting and consistency. There is pressure on the annotation staff from authors, the user community, and government funding agencies to sustain the current curation level. The goal is to have as small a backlog of structures as possible. Hiring more curators is not a viable, long-term solution to this challenge. However, annotation of structures deposited into the PDB can never be completely divorced from manual annotation. 3-D molecular objects are complex, and software tools that would exclude incorrect depositions with acceptable specificity and sensitivity are not possible in the short term. It also takes time to develop procedures and protocols for new or unusual types of data.

We believe that the solution lies in closer collaboration between the groups involved; the authors, database staff, and users of the PDB. From the curator's perspective, open exchange with programming groups outside the wwPDB would be helpful. Many widely used programs in structural biology are inconsistent on basic format requirements specified years ago [5]. Potential benefits from standardization range from increased automation of the deposition process to a more richly annotated dataset. In some cases, relatively simple changes in programs can have large impact on consistency of the data. We recognize that to be successful, this advice must be followed from within as well. Flexibility in data formats and improved, corrected, or new definitions of items in the database-format specifications could simplify requirements for scientific software development.

A good example of the need for greater communication is reflected in the basic format used for PDB files. The PDB flat file format is more than 35 years old, and is a poor container for the demands of modern bioinformatics. While the database infrastructure at the PDB ceased its dependence on this format years ago, almost all molecular structure programs still insist on PDB-formatted files. New formats that address most of the shortcomings of the PDB format, such as mmCIF [6] and XML [7], are available, but adoption by the general community has been slow.

It is clear from our experience that coordinating the efforts of biological and computer scientists is a complicated, time-consuming process that requires mutual respect and patience. One example of a successful RCSB PDB software project is pdb_extract [8], a program that extracts data needed for deposition from various crystallographic programs, reducing the human effort required to assemble complete and validated protein structure. Though relatively new, pdb_extract already has a positive impact on deposition and annotation by converting depositions into the preferred PDB archival format, mmCIF. Another RCSB PDB software development useful before and during data deposition is the web service Ligand Depot [9], which serves as a versatile tool for checking and building ligands. Authors are strongly encouraged to use the Validation Suite and Server [10] for structure validation before deposition.

To help long-term consistency of the data processing, the RCSB PDB has developed an annotation manual to describe curation protocols and include specific examples of difficult data, thus ensuring standardized curation practices among team members. The manual is publicly available (http://deposit.rcsb.org). The wwPDB is currently working on the challenge of standardizing curation guidelines across its centers. The RCSB PDB has been through a round of data uniformity in 2000 [11,12] and is currently undergoing another remediation effort with the wwPDB members.

### Integration with other resources.

The paradox with most databases, including the PDB, is that the current depth of annotation is both insufficient and overwhelming. The key is integration with other databases of biological properties. In this context, the challenges are 2-fold: identifying the “best” database to use, and standardizing the way data is exchanged between the various sites. Engaging the scientific community to reach a consensus is essential to solving these problems.

Last, could curators of the PDB improve the process of editing journal papers? The current editorial process does not include interaction with the PDB staff other than for a few journals providing citation information and checking availability of the structure at the time of publication. The quality of both the PDB and the papers would benefit from closer collaboration. As the PDB was formed with an archival mission, many would balk at the prospect of allowing the wwPDB to reject or to raise objections to structures. However, it also seems short-sighted not to utilize the talents of editorial scientists who are experts at analysis of molecular structure.

## Conclusion

The biocurator's success depends on many people; authors, programmers, and the users. Only discussion and collaboration among all parties involved can lead to the desired outcome: correctly annotated structures. Annotators and other wwPDB staff must continue to educate the community on the tools available and on deposition procedures to provide the community with the highest quality annotated data. 

## Abbreviations

PDB: Protein Data Bank
PDBj: Protein Data Bank—Japan
MSD–EBI: Molecular Structure Database—European Bioinformatics Institute
RCSB: Research Collaboratory for Structural Bioinformatics
wwPDB: worldwide PDB
